# Association of the combination of corporal adiposity and cardiorespiratory fitness with cardiometabolic risk factors in children — PREVOI Study

**DOI:** 10.1590/1984-0462/2025/43/2024105

**Published:** 2025-01-17

**Authors:** Renata Chácara Pires, Haysla Xavier Martins, Míriam Barbosa, Maria del Carmen Bisi Molina

**Affiliations:** a Universidade Federal do Espírito Santo, Programa de Pós-Graduação Nutrição e Saúde, Vitória, ES, Brazil.; b Universidade Federal do Espírito Santo, Programa de Pós-Graduação em Saúde Coletiva, Vitória, ES, Brazil.

**Keywords:** Sedentary behavior, Cardiovascular diseases, Risk factors for heart disease, Pediatric obesity, Public health, Comportamento sedentário, Doenças cardiovasculares, Fatores de risco de doenças cardíacas, Obesidade pediátrica, Saúde pública

## Abstract

**Objective:**

To assess the association between the combination of corporal adiposity (CA) and cardiorespiratory physical fitness (CRF) with cardiometabolic risk factors in children aged 7–10 years.

**Methods:**

Cross-sectional observational study with a sample of 251 children registered in Family Health Units. Sociodemographic, lifestyle, anthropometric, biochemical, blood pressure, and CRF data were collected. Cardiometabolic risk factors assessed: total cholesterol, HDL-c, LDL-c, triglycerides (TG), fasting glucose and blood pressure. CRF was assessed by the 6-minute run/walk test and classified into: “physically unfit” and “physically fit”. Nutritional status was assessed by body mass index (BMI)/age and categorized into CA groups: “no excess weight [≤ z-score+1]” and “excess weight [> z-score+1]”. CRF and CA were combined, and the children were classified as “no excess weight + physically fit”, “no excess weight + physically unfit”, “excess weight + physically fit” and “excess weight + physically unfit”. Bivariate analyses were performed, and Poisson regression models were tested. The Statistical Package for the Social Sciences (SPSS) version 21.0 software was used, adopting p<0.05.

**Results:**

Around 65% of the children had low CRF and 59% had excess weight (overweight+obesity). After adjustment, there was a greater occurrence of having altered HDL-c, TG and presence of ≥ 3 grouped cardiometabolic factors among those who had excess weight + physically unfit.

**Conclusions:**

The prevalence of altered HDL and TG and of ≥3 grouped cardiometabolic risk factors was significantly higher among children who had excess weight and were physically unfit.

## RESUMO

**Objetivo:**

Avaliar a associação entre a combinação da adiposidade corporal (AC) e da aptidão física cardiorrespiratória (ApFCR) com fatores de risco cardiometabólico em crianças (7–10 anos).

**Métodos:**

Estudo transversal com amostra de 251 crianças cadastradas em Unidades Saúde da Família. Dados sociodemográficos, de hábitos de vida, antropométricos, bioquímicos, de pressão arterial (PA) e ApFCR foram coletados. Fatores de risco cardiometabólico avaliados: colesterol total, HDL-c, LDL-c, triglicerídeos (TG), glicemia de jejum e pressão arterial (PA). A ApFCR foi avaliada pelo teste de corrida/caminhada de 6 minutos e classificada nos grupos “fisicamente inapto” e “fisicamente apto”. O estado nutricional foi avaliado pelo índice de massa corporal (IMC)/idade e categorizado em grupos de AC: “sem excesso de peso [≤z-score+1]” e “com excesso de peso [>z-score+1]”. AC e ApFCR foram combinados e as crianças foram categorizadas em: “sem excesso de peso+aptidão física”, “sem excesso de peso+inaptidão física”, “excesso de peso+aptidão física” e “excesso de peso+inaptidão física”. Análises bivariadas foram realizadas e modelos de regressão de Poisson testados, utilizado o software Statistical Package for the Social Sciences (SPSS) versão 21.0, adotando-se p<0,05.

**Resultados:**

Cerca de 65% da amostra apresentou baixa ApFCR e 59% excesso de peso. Foram observadas maiores ocorrências de HDL-colesterol alterado, TG alterado e da presença de ≥3 fatores cardiometabólicos agrupados em crianças com excesso de peso + inaptidão física.

**Conclusões:**

A prevalência de HDL e TG alterado e de ≥3 fatores cardiometabólicos agrupados foi significativamente maior entre as crianças com excesso de peso e que também apresentavam inaptidão física.

## INTRODUCTION

The significant increase in obesity in children is a public health problem, which according to the World Health Organization (WHO) can cause harm at any stage of life.^1^ Studies suggest that excess body adiposity during childhood and adolescence is the main risk factor for developing cardiometabolic problems in adulthood.^2,3^ In addition, changes in adiposity during childhood can predict the clustering of cardiometabolic risk factors during adolescence.^4,5^

Behavioral habits, such as physical inactivity and low physical fitness, play a significant role in this scenario.^4^ A systematic review and meta-analysis demonstrated that a low level of physical activity, low cardiorespiratory physical fitness (CRF), and sedentary behavior, represented by screen time >2 hours/day on weekends, were significantly associated with the development of metabolic syndrome in adolescence.^6^

In contrast, lifestyle behaviors aimed at physical activity and exercise, which contribute to improving CRF, can play a significant role in achieving a healthy cardiometabolic profile in children and adolescents.^2,7^ This is because achieving an adequate CRF is just as important as preventing excess body weight,^8,9^ and is inversely associated with cardiometabolic risk factors.^5,10^ Furthermore, good CRF can mitigate the negative effects of cardiometabolic risk factors associated with excess weight.^5,8^ However, these associations are not fully understood, especially in children.

To better understand the associaton between corporal adiposity, CRF, and cardiometabolic risk factors, and, given the existing gap, a study was carried out with children aged 7–10 years from a metropolitan region of Brazil. Our hypothesis is that children with excess weight who have a good CRF have a lower cardiometabolic risk compared to those who have a low CRF. Therefore, this study aimed to assess the association between the combination of excess weight and CRF with cardiometabolic risk factors in children aged 7 to 10 years.

## METHOD

Cross-sectional study with a sample of children (7–10 years) participating in the intervention study entitled “Prevention of Childhood Obesity in Primary Health Care: A community trial in the Metropolitan region of Vitória/ES”, which included four cities: Cariacica, Vitória, Vila Velha e Serra. The study was approved by the Ethics Committee for Research with Human Beings (UFES), under process number 4,581,297. All children signed the Assent Form and adults signed the Informed Consent Form.

The coverage of the Family Health Strategy (FHS) in the Family Health Units (FHU) varied from 25% (Cariacica) to 78% (Vitória). The estimated number of children aged 7–10 years registered at 82 FHU in these municipalities was 43,953. To calculate the sample for the main study, we considered a significant difference of 10% between the intervention and control groups, with type I error of 5% and type II error of 20%. Considering these parameters, the minimum sample size was 286. To this value, 20% was added, due to possible losses and refusals. Invitation to participate in the study was made by community health agents to all families with children aged 7–10 years of the FHU in the municipalities. Children of the same family could participate in the project. The sample consisted of 469 children (31.7%), distributed across 26 FHU. The highest participation occurred in Vitória (n=181) and the lowest in Cariacica (n=77), numbers that are proportional to the municipalities’ FHU coverage.

**Figure 1:**
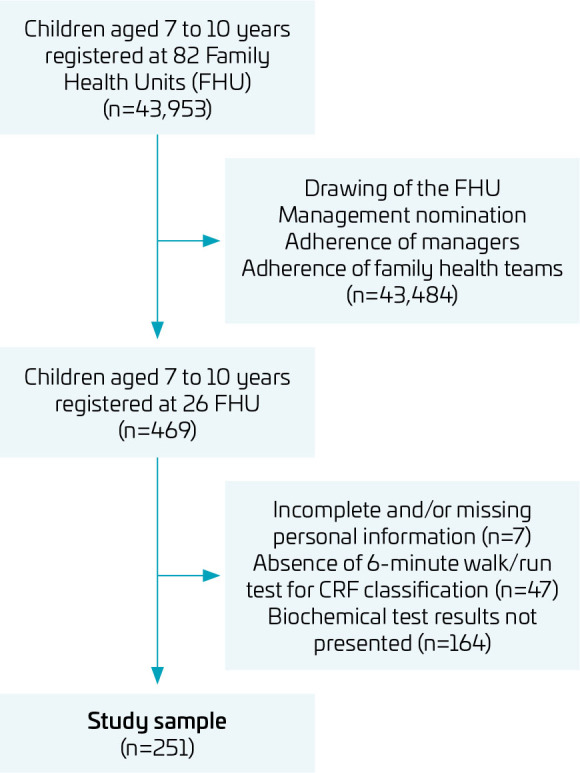
Flowchart of the sample selection process.

Source: prepared by the authors.

Therefore, children aged 7–10 years registered in 26 FHU were included, who had no cognitive, clinical, and/or physical limitations, as per medical records or reports by the primary caregiver, that would compromise the understanding of the study and make it impossible to perform the physical fitness test. Of the total number of participating children, seven presented incomplete and/or missing personal information, 47 did not complete the 6-minute walk/run test, and 164 did not present the results of the biochemical examination, all being excluded from the analyses of the present study. The final sample had 251 children (Figure 1).

Data collection took place from July to December 2021. Clinical examinations and interviews were carried out by trained and certified staff, on the premises of the FHU, based on prior scheduling of participants.

### Combination of corporal adiposity and cardiorespiratory fitness (independent variable)

Weight and height were measured using the protocol by Lohman et al.^11^ and body mass index (BMI) was calculated using the formula BMI=weight/height^2^ (kg/m^2^). Nutritional status was classified according to BMI, using the AnthroPlus™ tool, version 1.0.4, according to the WHO growth curves,^12^ into: thinness (< z-score -2), eutrophy (≥ z-score -2 and ≤ z-score + 1), overweight (> z-score +1 and ≤ z-score z +2), and obesity (>z-score +2). For analysis purposes, children were categorized into two CA groups: no excess weight (≤ z-score + 1) and excess weight (> z-score +1).

CRF was assessed using the 6-minute run/walk test, following criteria from the Sport Brazil Project (PROESP-BR) manual, by sex and age, which classifies children in the health risk zone (low CRF) and healthy zone (good CRF).^13^ The critical values by age and sex were: ≤730 m [7 years old boys] and ≤683 m [7 years old girls]; ≤768 m [8 years old boys] and ≤715 m [8 years old girls]; ≤820 m [9 years old boys] and ≤745 m [9 years old girls]; ≤856 m [10 years old boys] and ≤790 m [10 years old girls].^13^

The combination of CA with CRF created a variable with four categories, which were used as an independent variable:

1. No excess weight + physically fit;
2. No excess weight + physically unfit;
3. Excess weight + physically fit; and (4) excess weight + physically unfit.

### Cardiometabolic Risk Factors (dependent variable)

Biochemical data collection took place at the FHU, after a 10–12 hour fasting, by a health professional from the unit itself. Guidelines from the Brazilian Diabetes Society^14^ were used to assess fasting glycemia (GLY) and the Update of the Brazilian Guideline on Dyslipidemias and Prevention of Atherosclerosis^15^ for total cholesterol (TC), HDL cholesterol (HDL-c), LDL cholesterol (LDL-c), and triglycerides (TG). Blood pressure (BP) was measured three times on the right arm using a cuff suitable for the circumference of the arm, with a validated automatic device (OMROM™), after a 5-minute rest and a one-minute interval between measurements. The first measurement was discarded and the average of the other two was calculated. BP classification was carried out according to percentile tables,^16^ considering height, age, and sex. BP was classified as altered (elevated and hypertension) when systolic blood pressure (SBP) and/or diastolic blood pressure (DBP) were ≥ 90^th^ percentile. The six metabolic factors evaluated were dichotomized into “altered” and “not altered” according to the cutoff points already mentioned, and subsequently grouped into “<3 grouped cardiometabolic risk factors” and “≥3 grouped cardiometabolic risk factors”.

### Covariables

A semi-structured questionnaire was applied to obtain sociodemographic information about the family (family income, education of the head of the family, primary caregiver’s self-reported skin color [White, Black, and Brown]) and the child’s lifestyle (screen time, physical activity, and sleep habits).

Family income was obtained based on the criteria of the Brazilian Association of Research Companies (ABEP)^17^ and then grouped into “up to 1 minimum wage”, “from 1 to 3 minimum wage”, and “above 3 minimum wages”. The education level of the primary caregiver was grouped into “illiterate/incomplete elementary school”, “complete elementary school”, “high school education”, and “higher education”.^17^

Sedentary behavior was analyzed based on the total daily screen time — ST (television, video games, cell phones, and computers). Using the recommendation of the Brazilian Society of Pediatrics,^18^ children were classified as “adequate” (screen time ≤120 minutes) and “inadequate” (screen time >120 minutes).

For the analysis of physical activity, 300 minutes per week was adopted as a cutoff point, considering the minimum of 60 minutes per day recommended for children.^19^ Only sports and leisure activities were considered, excluding physical education classes, commuting, work, or housework. Participants were classified as “active” or “insufficiently active”.

Sleep habits were classified according to the National Sleep Foundation, which recommends 10 to 11 hours/night (adequate), and not recommended (inadequate) less than seven hours and more than 12 hours of sleep for the age group of 6–10 years.^20^

Descriptive analyses of sociodemographic variables and lifestyle habits and cardiometabolic risk factors were carried out, data were expressed as n (%) and mean ± standard deviation. To assess data normality, the Kolmogorov-Smirnov test was used. Symmetrical continuous variables were described as mean and standard deviation, and asymmetric variables as median and interquartile range (p25 to p75). Pearson’s chi-square test (X^2^) or Fisher’s exact test were used for categorical variables and Student’s t-test or Mann-Whitney test for quantitative variables.

Poisson regression models with robust variance were used to test associations between independent variable (combination of CA + CRF) and outcomes (cardiometabolic risk factors and grouping of factors). We included in the regression only the risk factors and covariables that presented p≤0.20 in the bivariate analysis (for the covariables, statistical significance should be observed for both exposure and outcomes). Therefore, specific adjustment models were created for each outcome variable. The respective prevalence ratio (PR) and confidence interval (95% CI) values were then presented, with statistical significance established at p<0.05. For all of them, the assumptions of absence of multicollinearity, minimum sample size for the number of variables in the model, and absence of outliers were tested. Statistical analyses were performed using the IBM Statistical Package for the Social Sciences (SPSS) Statistics 21.0 software (Armonk, NY: IBM Corp).

## RESULTS

A total of 251 children were evaluated, with a mean age of 8.57±1.12 years, and mostly girls (53.8%). The majority belonged to families earning 1–3 minimum wages (63.5%), with the head of the family having high school education (50.0%) and the primary caregiver self-declaring to be Brown (59.0%).

The prevalence of insufficiently active children was 46.2% (n=116), with a higher porcentage among girls (n=64; 47.4%). As for CRF, 65.3% (n=164) were in the risk zone, and this number was higher in boys (n=84; 72.4%). Inadequate ST was 87.6% (n=220) and around 75% (n=189) of the children had inadequate sleep hours, this being higher in boys (81.9%, n=95). We identified a significant difference between the sexes only for family income (p=0.042), CRF (p=0.029), and sleep habits (p=0.025) (data not presented in tables).

Table 1 shows the combinations of CA (without and with excess weight) + CRF, according to cardiometabolic risk factors and metabolic grouping. A significant percentage of the children with altered HDL-c, altered TG, and with ≥3 grouped cardiometabolic risk factors were excess weight + physically unfit (63.3, 61.3, and 67.6% [p<0.001], respectively).

**Table 1:** Combination of corporal adiposity and cardiorespiratory physical fitness, according to cardiometabolic risk factors in children aged 7 to 10 years registered in Family Health Units. PrevOI Study (n=251).

CA + CRF	Cardiometabolic risk factors*						Grouped cardiometabolic risk factors	
	Altered GLY	Altered TC	Altered HDL-c	Altered LDL-c	Altered TG	Altered BP		
							<3 factors	≥3 factors
No excess weight + physically fit	2 (28.6)	21 (20.8)	14 (15.6)	12 (17.9)	19 (16.0)	9 (14.1)	47 (25.7)	9 (13.2)
No excess weight + physically unfit	2 (28.6)	17 (16.8)	8 (8.9)	10 (14.9)	10 (8.4)	8 (12.5)	43 (23.5)	4 (5.9)
Excess weight + physically fit	-	12 (11.9)	11 (12.2)	10 (14.9)	17 (14.3)	10 (15.6)	22 (12.0)	9 (13.2)
Excess weight + physically unfit	3 (42.9)	51 (50.5)	57 (63.3)	35 (52.2)	73 (61.3)	37 (57.8)	71 (38.8)	46 (67.6)
p-value	0.730^†^	0.783^‡^	**<0.001** ^‡^	0.456^‡^	**<0.001** ^‡^	0.059^‡^	**<0.001** ^‡^	

CA: corporal adiposity; CRF: cardiorespiratory physical fitness; GLY: fasting glycemia (altered ≥100 mg/dL); TC: total cholesterol (altered ≥170 mg/dL); HDL-c: high-density lipoprotein (altered ≤45 mg/dL); LDL-c: low-density lipoprotein (altered ≥110 mg/dL); TG: triglycerides (altered ≥90 mg/dL); BP: blood pressure (altered when systolic blood pressure and/or diastolic blood pressure are≥90^th^ percentile indicating high blood pressure and ≥95^th^ percentile indicating hypertension, according to age, sex, and height percentile). Values expressed in n (%).

*Comparison category omitted: not altered;

^†^Fisher’s Exact Test; Statistically significant values in bold;

^‡^Chi-square test.

The sociodemographic and lifestyle variables according to the combination of CA + CRF and according to cardiometabolic risk factors are found in Tables 2 and 3, respectively. Around 54% of the excess weight + physically unfit children did not meet physical activity recommendations (p=0.010). Furthermore, 52.9% of those with altered TG were also insufficiently active (p=0.042).

**Table 2:** Sociodemographic and lifestyle variables according to the combination of corporal adiposity and cardiorespiratory physical fitness in children aged 7 to 10 years registered in Family Health Units. PREVOI Study (n=251).

Variables	No excess weight + physically fit	No excess weight + physically unfit	Excess weight + physically fit	Excess weight + physically unfit	p-value
Sex					
Masculine	17 (30.4)	25 (53.2)	15 (48.4)	59 (50.4)	0.058*
Feminine	39 (69.6)	22 (46.8)	16 (51.6)	58 (49.6)	
Age (years)					
7	17 (30.4)	8 (17.0)	6 (19.4)	27 (23.1)	0.245^†^
8	13 (23.2)	9 (19.1)	11 (35.5)	27 (23.1)	
9	10 (17.9)	17 (36.2)	10 (32.3)	27 (23.1)	
10	16 (28.6)	13 (27.7)	4 (12.9)	36 (30.8)	
Family income (minimum wage)^‡^					
Up to 1	4 (7.1)	10 (22.2)	2 (6.5)	18 (15.4)	0.103^†^
1–3	41 (73.2)	25 (55.6)	17 (54.8)	75 (64.1)	
>3	11 (19.6)	10 (22.2)	12 (38.7)	24 (20.5)	
Education of the head of the family^‡^					
Illiterate/incomplete elementary school	11 (21.6)	8 (21.1)	10 (32.3)	23 (20.5)	0.477^†^
Complete elementary school	13 (25.5)	4 (10.5)	2 (6.5)	17 (15.2)	
High school education	23 (45.1)	21 (55.3)	14 (45.2)	58 (51.8)	
Higher education	4 (7.8)	5 (13.2)	5 (16.1)	14 (12.5)	
Primary caregiver’s self-reported skin color^‡^					
White	6 (10.7)	8 (17.8)	11 (35.5)	24 (20.5)	0,066*
Black	15 (26.8)	6 (13.3)	8 (25.8)	24 (20.5)	
Brown	35 (62.5)	31 (68.9)	12 (38.7)	69 (59.0)	
Adequate screen time (≤2 h/day)	9 (16.1)	9 (19.1)	4 (12.9)	9 (7.7)	0.140^†^
Physically active (≥ 1 hour/day)	40 (71.4)	22 (46.8)	19 (61.3)	54 (46.2)	0.010*
Adequate sleep habit	19 (33.9)	9 (19.1)	8 (25.8)	26 (22.2)	0.290*
BMI/age (score)	-0,16±0,8^a^	-0,38±1,1^a^	2,0±0,7^b^	2,6±1,2^b^	**<0,001** ^§^

BMI: Body Mass Index. Values expressed in n(%).

*Chi-square test;

^†^Fisher’s Exact Test;

^‡^n different due to lack of data;

^§^Kruskal-Wallis’s test (followed by post hoc pairwise — same letters do not differ from each other, different letters differ from each other). Statistically significant values in bold.

**Table 3:** Sociodemographic and lifestyle variables according to cardiometabolic risk factors* in children aged 7 to 10 years registered in Family Health Units. PREVOI Study (n=251).

Variables	Altered HDL-c^†^	p-value	Altered TG^a^	p-value	Altered BP^†^	p-value	≥3 grouped cardiometabolic risk factors^†^	p-value
Sex								
Masculine	40 (44.4)	0.961^‡^	50 (32.0)	0.205^‡^	27 (42.2)	0.454^‡^	28 (41.2)	0.329^‡^
Feminine	50 (55.6)		69 (58.0)		37 (57.8)		40 (58.8)	
Age (years)								
7	22 (24.4)	0.125^‡^	29 (24.4)	0.591^‡^	17 (26.6)		25 (36.8)	**0.001** ^‡^
8	18 (20.0)		29 (24.4)		10 (15.6)		6 (10.0)	
9	30 (33.3)		33 (27.7)		19 (29.7)		20 (31.3)	
10	20 (22.2)		28 (23.5)		18 (28.1)		17 (25.0)	
Family income (minimum wage)								
Up to 1	12 (13.3)	0.936^‡^	14 (11.8)	0.350^‡^	13 (20.3)	0.173^‡^	8 (11.8)	0.808^‡^
1–3	57 (63.3)		81 (68.1)		36 (56.3)		43 (63.2)	
>3	21 (23.3)		24 (20.2)		15 (23.4)		17 (25.0)	
Education of the head of the family								
Illiterate/incomplete elementary school	20 (23.5)	0.803^‡^	25 (22.7)	0.517^‡^	15 (25.0)	0.687^‡^	11 (17.2)	0.297^‡^
Complete elementary school	10 (11.8)		21 (19.1)		7 (11.7)		8 (12.5)	
High school education	43 (50.6)		52 (47.3)		32 (53.3)		34 (53.1)	
Higher education	12 (14.1)		12 (10.9)		6 (10.0)		11 (17.2)	
Primary caregiver’s self-reported skin color								
White	24 (10.7)	0.172^‡^	27 (22.7)	0.389^‡^	11 (17.2)	0.653^‡^	15 (22.1)	0,661^‡^
Black	21 (23.3)		22 (18.5)		16 (25.0)		16 (23.5)	
Brown	45 (50.0)		70 (58.8)		37 (57.8)		37 (54.4)	
Adequate screen time (≤2 h/day)	11 (12.2)	0.705^‡^	10 (8.4)	0.071^‡^	6 (9.4)	0.402^‡^	7 (10.3)	0.546^‡^
Physically active (≥1 hour/day)	48 (53.3)	0.481^‡^	56 (47.1)	**0.042** ^‡^	32 (50.0)	0.482^‡^	35 (51.5)	0.654^‡^
Adequate sleep habit	23 (25.6)	0.878^‡^	29 (24.4)	0.908^‡^	11 (17.2)	0.106^‡^	16 (23.5)	0.793^‡^

Values expressed in n (%).

*only factors with p≤ 0.20 in Table 1;

^†^Comparison category omitted: not altered/ <3 cardiometabolic factors;

^‡^Chi-square test. Statistically significant values in bold.

HDL-c: high-density lipoprotein (altered ≤45 mg/dL); TG: triglycerides (altered ≥90 mg/dL); BP: blood pressure (altered when systolic blood pressure and/or diastolic blood pressure ≥90^th^ percentile, according to age, sex and height percentile).

Table 4 presents the respective PR (95%CI) for each altered cardiometabolic risk factor and grouping of ≥3 grouped cardiometabolic risk factors, according to the combination of CA + CRF. After adjustment for possible confounding variables, there was a greater occurrence of having altered HDL-c, TG and presence of ≥3 grouped cardiometabolic risk factors among those who had excess weight + physically unfit (PR 1.98 [95%CI 1.22–3.21]; PR 1.76 [95%CI 1.17–2.64]; PR 2.52 [95%CI 1.37–4.65], respectively).

**Table 4:** Prevalence ratio and 95% confidence interval (95%CI) of cardiometabolic risk factors according to the combination of corporal adiposity and cardiorespiratory physical fitness in children aged 7 to 10 years registered in Family Health Units. PREVOI Study (n=251).

CA + CRF	Cardiometabolic risk factors									≥3 grouped cardiometabolic risk factors^§^		
	Altered HDL-c*			Altered TG^†^			Altered BP^‡^					
	p-value	PR	95%CI	p-value	PR	95%CI	p-value	PR	95%CI	p-value	PR	95%CI
No excess weight + physically fit		1			1			1			1	
No excess weight + physically unfit	0.537	0.78	0.36–1.68	0.166	0.62	0.32–1.20	0.987	1.00	0.42–2.36	0.273	0.53	0.17–1.63
Excess weight + physically fit	0.444	1.29	0.66–2.53	0.057	1.62	0.98–2.66	0.098	1.97	0.88–4.42	0.085	1.99	0.90–4.38
Excess weight + physically unfit	**0.005**	**1.98**	**1.22–3.21**	**0.006**	**1.76**	**1.17–2.64**	0.063	1.86	0.96–3.60	**0.003**	**2.52**	**1.37–4.65**

CA: corporal adiposity; CRF: cardiorespiratory physical fitness; HDL-c: high-density lipoprotein; TG: triglycerides; BP: blood pressure; PR: prevalence ratio.

*Reference: adequate HDL-c (>45 mg/dL); Adjusted model: primary caregiver’s self-reported skin color;

^†^Reference: adequate TG (<90 mg/dL); Adjusted model: sex, physical activity, screen time;

^‡^Reference: adequate BP (when systolic blood pressure and/or diastolic blood pressure are lower than the 90^th^ percentile, according to age, sex and height percentile); Adjusted model: family income;

^§^Reference: <3 grouped cardiometabolic factors; Model adjusted for: age.

It is worth noting that, although there was no statistical significance observed for BP in the dichotomous variable (“altered” and “not altered”), the mean test indicated differences in DBP and SBP values between the CA + CRF combination categories (data not presented in tables). Children with the worse combination (excess weight + physically unfit) presented higher means of DBP (66.9±10.8 mmHg vs. 61.9±10.4 mmHg; p=0.002) and SBP (105.9±11.3 mmHg vs. 100.6±9.4 mmHg; p=0.003) when compared to the best combination (no excess weight + physically fit) (data not presented in tables).

## DISCUSSION

In this study, the combination of excess weight + physically unfit in children aged 7–10 years increases the occurences of some studied cardiometabolic risk factors, such as altered HDL-c and TG, and the presence of ≥3 grouped cardiometabolic risk factors.

Studies indicate an association between low CRF and higher cardiometabolic risk,^5,21,22^ which corroborates the findings of the present study. Although excess weight remains a fundamental factor in the associations with cardiometabolic parameters, the influence of CRF is notably pronounced in reducing the prevalence of these altered parameters.

Better CRF can reduce cardiometabolic risk through mechanisms that possibly involve genetic aspects, adipocytokines, and oxidative capacity of skeletal muscles, even in overweight/obese children.^23^ The positive influence of CRF extends to multiple physiological systems and can improved insulin action and glucose transport, optimize fat metabolism, increased HDL cholesterol levels, and decreased sympathetic tone and blood pressure.^24^ These wide-ranging benefits of CRF are particularly relevant during childhood, a critical period for developing healthy lifestyle habits and preventing chronic diseases in adulthood. Thus, the association between CRF and cardiometabolic risk factors justify the inclusion of CRF assessment as an integral component of children’s health monitoring. This practice would facilitate early and more effective interventions, contributing to reducing the risk of cardiometabolic diseases in the pediatric population.

Similar to the results found in our study, a survey^23^ carried out with children (7–9 years old), which used a combination of BMI and CRF, showed that the group classified as eutrophic and with good CRF had a lower score for the development of metabolic syndrome, while overweight children with low CRF had a higher score.^23^ Such evidence signals a less favorable metabolic profile in overweight plus physically unfit children, suggesting that adequate levels of CRF may reduce the impact of BMI on the development of metabolic syndrome and improve metabolic profiles,^23,25^ indicating that CRF reduces overall cardiometabolic risk in children.^26^

In our study, although we did not observe statistical significance for the dichotomous variable blood pressure (“altered” and “not altered”), the test of means indicated significant differences in SBP and DBP values between the excess weight and physical fitness categories. Children with the worst combination (excess weight + physically unfit) had higher mean SBP and DBP than those with the best combination (no excess weight + physically unfit). These findings are in line with Tornquist et al.,^5^ who reported a higher prevalence of elevated SBP among overweight and obese schoolchildren, especially the physically unfit. Our findings, together with Tornquist et al.,^5^ highlight the importance of addressing both excess weight and physical fitness to reduce the risk of hypertension and other cardiovascular diseases in children.

In our study, more than half of the children had low CRF and insufficiently active children were more present in the excess weight + physically unfit category, suggesting that there is a relationship between CRF and physical activity. The reduction in physical activity in this population can also be attributed to the increase in sedentary behavior in front of screens, which ends up favoring the early development of overweight/obesity and chronic non-communicable diseases.^27^ Furthermore, it was identified that the occurrence of children with low levels of HDL-c, altered TG, and ≥3 grouped cardiometabolic risk factors were significantly higher in the excess weight + physically unfit category. These findings corroborate studies that highlight the concern about low physical fitness and its influence on cardiometabolic risk factors in children.^5,23^ Therefore, encouraging children to take part in physical activities that have a positive impact on the development of physical fitness is fundamental due to their close relationship with cardiometabolic risk factors, especially when CA is present,^23^ despite the limited research on children up to ten years of age.

Our findings demonstrate that a good CRF can reduce the prevalence of altered cardiometabolic outcomes in children with excess weight. Taking this scenario into account, developing strategies to improve CRF levels is of fundamental importance, especially in areas with less access to physical activity. Furthermore, there is evidence that good CRF levels during childhood result in a healthier cardiometabolic profile in adulthood^28^ as well as that, adult individuals with low levels of CRF had two to three times the risk of mortality from cardiovascular diseases at all BMI levels, when compared to eutrophic and physically fit individuals.^29^ However, the combined influence of CA and CRF is still limited in research with children.

As a limitation of the study, we highlight its cross-sectional design, which makes it impossible to establish a causal relationship between the variables, in addition to caution with the generalization of these data to other regions of the country due to possible ethnic, cultural, and socioeconomic differences. Another point is the possible influence of unmeasured factors such as sexual maturation, diet, and genetic factors, as cardiometabolic risk is a multifactorial issue. The use of BMI in the assessment of adiposity and the assessment of CRF levels using the 6-minute walk/run test, although widely used, especially in population assessments in studies with children,^5,30^ may be limiting points. Although the CRF test used is strongly correlated with the CRF measurement in the laboratory, motivational factors can affect the test result.^5,30^

In order to mitigate the effects of the limitations mentioned, and guarantee the quality of the data obtained, priority was given to the standardization of routines and consolidated procedures for carrying out the interview, measuring anthropometric measurements, and the 6-minute walk/run test, this being a strength of the present study. Furthermore, this research used the combination CA + CRF as an exposure variable, still little explored, and cardiometabolic risk factors as outcomes, in children registered at a FHU in a metropolitan region.

We concluded that the prevalence of low HDL, high TG and ≥3 grouped cardiometabolic risk factors was significantly higher among children who had excess weight and were physically unfit. These results suggest a protective role for CRF in mitigating the negative effects of excess weight on cardiometabolic parameters in children, reinforcing the importance of including CRF in child health assessments. Due to the inherent limitations of a cross-sectional study, it is suggested that future studies adopt a longitudinal design, allowing changes in CRF levels, CA and cardiometabolic risks to be monitored over time, as well as including an assessment of sexual maturity, controlling for variables such as dietary habits, genetic factors and inflammatory markers, providing a more comprehensive analysis of the factors that influence cardiometabolic risk, broadening the analysis of the information produced in this investigation.

## Data Availability

The database that originated the article is available with the corresponding author. CAAE: 39633320.0.0000.5060
